# Supplementary material for: Predicting metabolic adaptation from networks of mutational paths
Source: Nat Commun. 2017 Sep 25;8:685. doi: 10.1038/s41467-017-00828-6 (PMC5612958; doi:10.1038/s41467-017-00828-6)
Supplement: Supplementary file 1 — Supplementary Information [file 41467_2017_828_MOESM1_ESM.pdf]

Supplementary Note 1. Schematics of the model when a cell's phenotype is interpreted as the density of cellular permeases

$$n_{i,x} + u \xrightarrow{k_u} \tilde{n}_{i,x} \begin{cases} \xrightarrow{\alpha_x} n^{(u)}_{i,x} \xrightarrow{m_u} \begin{cases} n_{i+\gamma_u,x} & i+\gamma_u \leq N_s \\ n_{i+\gamma_u-N_s,x} + n_{1,x} & i+\gamma_u > N_s \end{cases} \\ \xrightarrow{\beta_x} n_{i,x} + u \end{cases}$$

$$n_{i,x} + v \xrightarrow{k_v} \tilde{n}_{i,x} \begin{cases} \xrightarrow{\alpha_x} n_{i,x} + v \\ \xrightarrow{\beta_x} n^{(v)}_{i,x} \xrightarrow{m_v} \begin{cases} n_{i+\gamma_v,x} & i+\gamma_v \leq N_s \\ n_{i+\gamma_v-N_s,x} + n_1 & i+\gamma_v > N_s \end{cases} \end{cases}$$

$$\emptyset \xrightarrow{u^{(0)}} u$$

$$\emptyset \xrightarrow{v^{(0)}} v$$

$$\{n_{i,x}, n^{(u)}_{i,x}, n^{(v)}_{i,x}, u, v\} \xrightarrow{D} \emptyset$$

For the phenotype indexed by x, we parametrize the abundance of u-permeases by α_x, the abundance of v-permeases by β_x, and assume that the total abundance of permeases is fixed. Here $n_{i,x}$ is the abundance of cells with phenotype x in growth state i; $\tilde{n}_{i,x}$ is the abundance of cells in state i bound to a substrate; and $n^{(u)}_{i,x}$ is the abundance of cells that are actively metabolizing u and $n^{(u)}_{i,x}$ is the equivalent for v. Substrate u is encountered by cells at a rate proportional to k_u, and the probability that a u substrate binds a u-permease is proportional to the density of u-permeases on the cell: $s_x = \alpha_x/(\alpha_x + \beta_x)$. Once a substrate is encountered, we assume that the substrate is either imported or lost quickly so that $\tilde{n}_{i,x}$ is at quasi steady-state. When imported, a unit of substrate is metabolized at substrate-specific rate, m_u for u and m_v for v, and we assume that a cell cannot import more substrate until the previous unit is fully metabolized. Substrates u and v enter the chemostat at rates $u^{(0)}$ and $v^{(0)}$, and cells and substrates are washed out at a rate D.

Supplementary Note 2. Schematics of the model when a cell's phenotype is interpreted as the probability of stochastic switching between metabolic states

$$n_{i,x,u} + u \xrightarrow{k_u} n_{i,x}^{(u)} \xrightarrow{m_u} \begin{cases} \tilde{n}_{i+\gamma_u,x} & i+\gamma_u \leq N_s \\ \tilde{n}_{i+\gamma_u-N_s,x} + \tilde{n}_{1,x} & i+\gamma_u > N_s \end{cases}$$

$$n_{i,x,u} + v \xrightarrow{k_v} \tilde{n}_{i,x} + v$$

$$n_{i,x,v} + v \xrightarrow{k_v} n_{i,x}^{(v)} \xrightarrow{m_v} \begin{cases} \tilde{n}_{i+\gamma_v,x} & i+\gamma_v \leq N_s \\ \tilde{n}_{i+\gamma_v-N_s,x} + \tilde{n}_{1,x} & i+\gamma_v > N_s \end{cases}$$

$$n_{i,x,v} + u \xrightarrow{k_u} \tilde{n}_{i,x} + u$$

$$\tilde{n}_{i,x} \xrightarrow{\alpha_x} n_{i,x,u}$$

$$\tilde{n}_{i,x} \xrightarrow{\beta_x} n_{i,x,v}$$

$$\emptyset \xrightarrow{u^{(0)}} u$$

$$\emptyset \xrightarrow{v^{(0)}} v$$

$$\{n_{i,x,u}, n_{i,x,v}, n_{i,x}^{(u)}, n_{i,x}^{(v)}, u, v\} \xrightarrow{D} \emptyset$$

A cell is in a state that can use u substrate or a state that can use v substrate or is transitioning between states. Here $n_{i,x,u}$ is the abundance of cells with phenotype x in growth state i and in metabolic state u; $n_{i,x,v}$ is the abundance of cells with phenotype x in growth state i and in metabolic state v; $\tilde{n}_{i,x}$ is the abundance of cells transitioning from one state to another; and $n_{i,x}^{(u)}$ is the abundance of cells that are actively metabolizing u and $n_{i,x}^{(u)}$ is the equivalent for v. The phenotype value, $s_x = \alpha_x/(\alpha_x + \beta x)$, is the probability that a cell switches to the u-metabolic state, and $(1 - s_x)$ is the probability that the cell switches to the v-metabolic state. We assume that transitions are independent and that the switching probabilities are not affected by the environment. A cell can, however, transition state by encountering the other substrate. Cells that have imported a substrate cannot import more substrates until the current substrate has been metabolized, at rate m_u for u and m_v for v. Substrates u and v enter the chemostat at rates $u^{(0)}$ and $v^{(0)}$, and cells and substrates are washed out at a rate D.

Supplementary Note 3. Algorithm for constructing the invasion map

The algorithm exhaustively simulates all invasion models by dynamically identifying which invasion scenarios are relevant (an example is shown in Fig. 2). To reduce the number of equations during numerical integration, we also dynamically re-generated the chemostat model to include only the equations for populations with non-zero initial abundances. The algorithm accepts a set of nine environmental parameters as arguments and returns: *1.* A dictionary (an associative array) of dynamically-stable resident phenotype composition states, $\mathcal{C}$, arbitrarily indexed by order of discovery. *2.* An identically-keyed dictionary holding the abundances for the phenotype populations and substrates at steady-state, $\mathcal{N}$. *3.* An array of state transitions, T, where each element has the form (i, y, j) to indicate that composition state i is transformed to state j as a result of invasion by mutant phenotype y with phenotype value s_y. Pseudocode for the algorithm is shown below.

```
Initialize data structures (with phenotype-null state, indexed as 0)
𝒞 ← (0 : C_0 = [0, 0, ..., 0])                ▷ dictionary of unique resident phenotype compositions
𝒩 ← (0 : N_0 = [0, 0, ..., 0, u*, v*])         ▷ dictionary of chemostat steady states
T ← ∅                                          ▷ array of transition tuples, initially empty
Q ← 0                                          ▷ FIFO queue, initialized with state 0
$counter = 0                                   ▷ integer to keep track of number of unique composition states
Main loop constructs invasion map (tree) in breadth-first manner
while Q is not empty do
    i ← dequeue (Q)                            ▷ state index from front of queue
    C_i ← 𝒞[i]
    N_i ← 𝒩[i]
Challenge state with all novel phenotype mutants
    for (y = 0; y ≤ 10; y++) do
        if C_{i,y} == 1 then
            continue                           ▷ phenotype already exists in resident composition
        end if
        if (i, y, ·) ∈ T then
            continue                           ▷ mutation/invasion event simulated elsewhere
        end if
Must simulate mutation/invasion event
        construct model equations, M
        ϵ = δ · min_{N_{i,x}>0}{N_{i,x}}       ▷ size of nascent mutant population and extinction threshold
        perturb resident steady state to get initial conditions: N_{i,y} = N_{i,y} + ϵ
        integrate M numerically, with perturbed initial conditions, to steady state, N_k
Inspect steady state and remove extinct phenotypes:
        C_{k,x} = { 1  N_{k,x} > ϵ
                  { 0  otherwise
        N_{k,x} = { N_{k,x}  C_{k,x} == 1
                  { 0        otherwise
        if C_k ∉ 𝒞 then                        ▷ discovered a new state?
            j = ++$counter                     ▷ increment index counter for new state
            𝒞 ← (j : C_k)
            𝒩 ← (j : N_k)
            Q ← enqueue(j)                     ▷ enqueue the new state for future inspection at the back of the queue
        else
            j = k' s.t. 𝒞[k'] = C_k            ▷ reverse look-up to get index of already discovered state
        end if
        T ← (i, y, j)  ▷ append state transition (from i to j as a result of invasion by mutant y)
    end for
end while
```

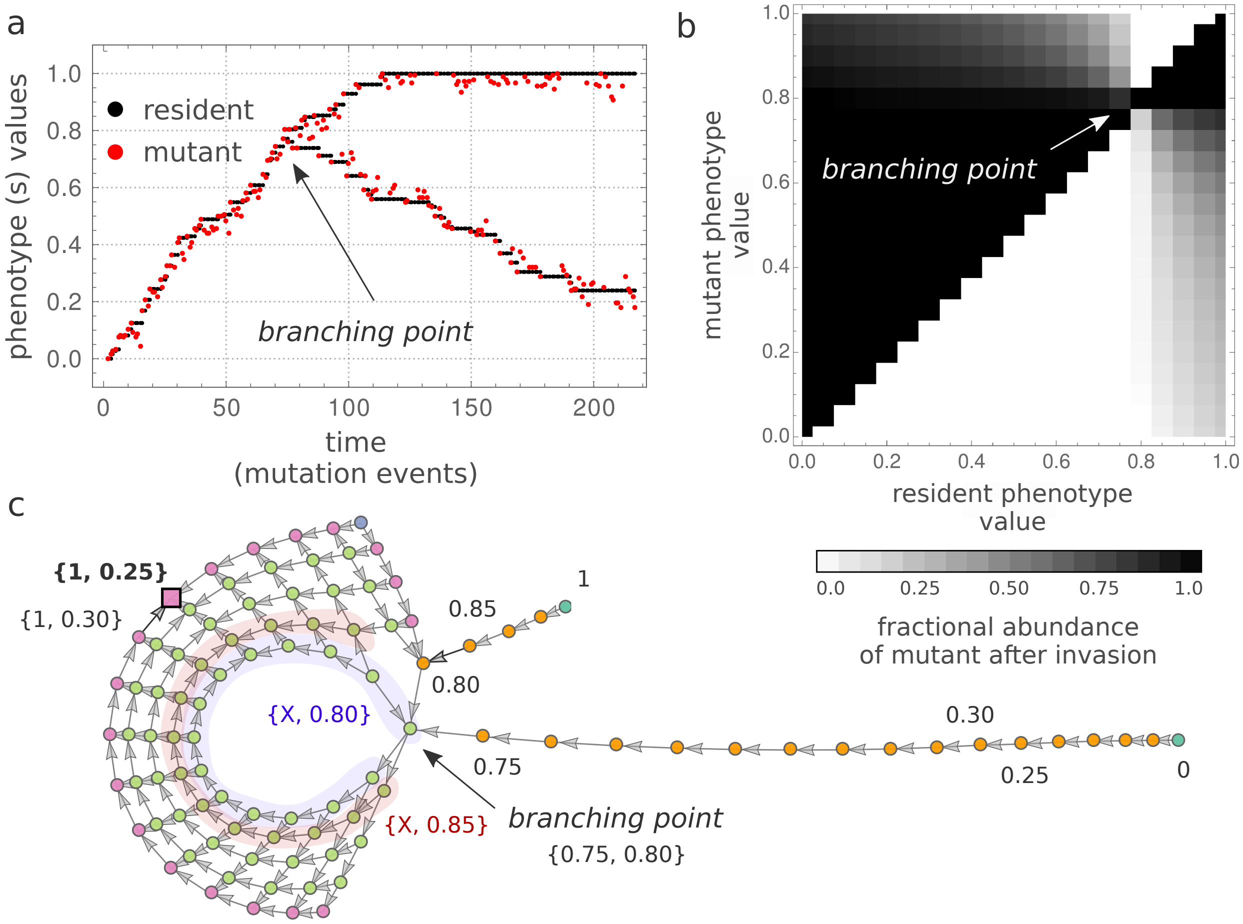

Supplementary Figure 1. Pairwise invasibility plots (PIPs) are not sufficient to describe the full repertoire of adaptation dynamics. We illustrate this insufficiency with an example that includes a branching event. (a) A sample mutational path in continuous phenotype space passes through a branching point where the monomorphic population becomes a dimorphic community. At each time point (representing a mutation), the black dot denotes the current residents' phenotypes and the red dot denotes the mutant's phenotype. If a mutant invades, the red dot becomes a black dot at the following time point. (b) The PIP shows the branching point and adequately describes monomorphic adaptation dynamics leading there. After branching, however, ecological dynamics are no longer pairwise (since there are two residents and one mutant) and the PIP cannot describe the subsequent trajectory seen in A that leads to the establishment of a specialist and a generalist. (c) The network of mutational paths reproduces the information contained in the pairwise invasibility plot (monomorphic adaptation dynamics in the network's tail), but also describes the fate of all possible transient communities after the branching point. To highlight the PIPs' insufficiency, we note two examples where the fate of a phenotypic mutant cannot be explained by pairwise interactions. A $s = 0.30$ mutant can invade a $s = 0.25$ resident but the converse is not possible. When the community consists of two resident phenotypes, $\{s_1 = 1, s_2 = 0.3\}$, however, a $s = 0.25$ mutant phenotype can invade and drive the $s = 0.30$ resident to extinction in disagreement with the competition outcomes predicted by the PIP. A similar reversal of invasibility occurs between $s = 0.80$ and $s = 0.85$, where the former can invade and drive the latter to extinction in the monomorphic setting, but the resident community $\{s_1 = X, s_2 = 0.80\}$ (blue highlight) can by invaded by a $s = 0.85$ mutant (red highlight) in the presence of certain other co-residents.

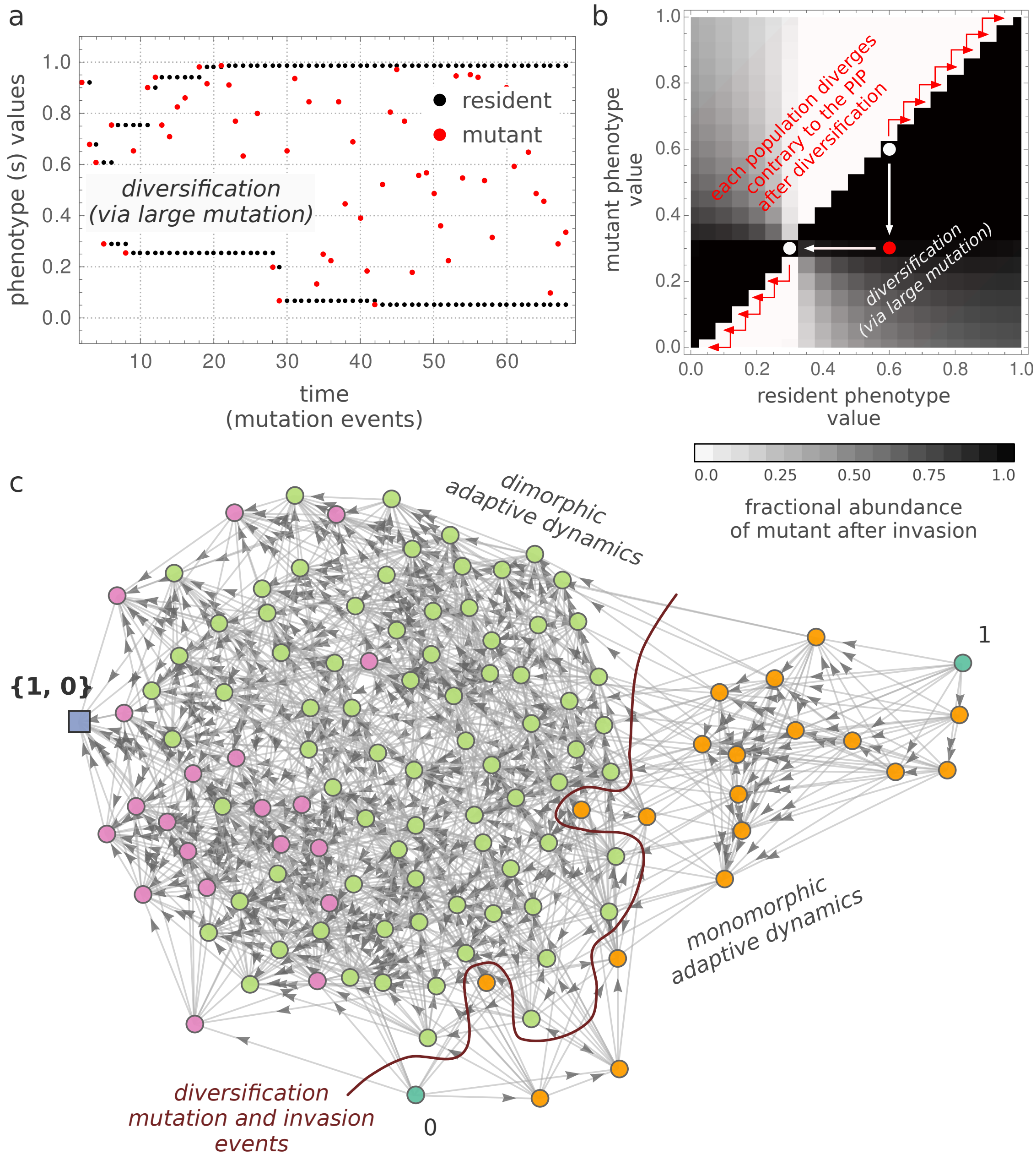

Supplementary Figure 2. Standard (local) adaptive dynamics theory is not sufficient for describing adaptation dynamics with large mutations. We illustrate this insufficiency with an example that includes large-effect mutations that induce population diversification and co-existence. (a) A sample mutational path in continuous phenotype space undergoes diversification via a large-effect mutation (monomorphic population with $s \approx 0.6$ resident becomes dimorphic after invasion by $s \approx 0.3$ mutant) after 4 mutations. The two co-residents then diverge to the evolutionarily stable community with two metabolic specialists. (b) The PIP shows that the branching point around $s \approx 0.3$ is convergence-stable in the limit of infinitesimally-small mutations: monomorphic populations where $s < 0.3$ or $s > 0.3$ will converge to the branching point. A local invasion analysis of the branching point will suggest that it is not an ESS because nearby mutants can invade. However, the fate of the dimorphic community after branching cannot be understood from the PIP — in fact, subsequent divergence proceeds *contrary* to the information in the PIP. In addition, when mutations have large-effect, there are many possible mutation and invasion events that lead to diversification, as indicated by the grey bands, and a local analysis of the branching point is insufficient. (c) The network of mutational paths (for intermediate-size mutations) visualizes both monomorphic and dimorphic adaptation dynamics. We highlight the existence of many diversification mutation and invasion events (edges crossing the red curve), most of which involve large-effect mutations. The resulting transient resident-mutant communities cannot be analyzed by standard adaptive dynamics theory, which assumes a continuum of infinitesimally-small mutations.

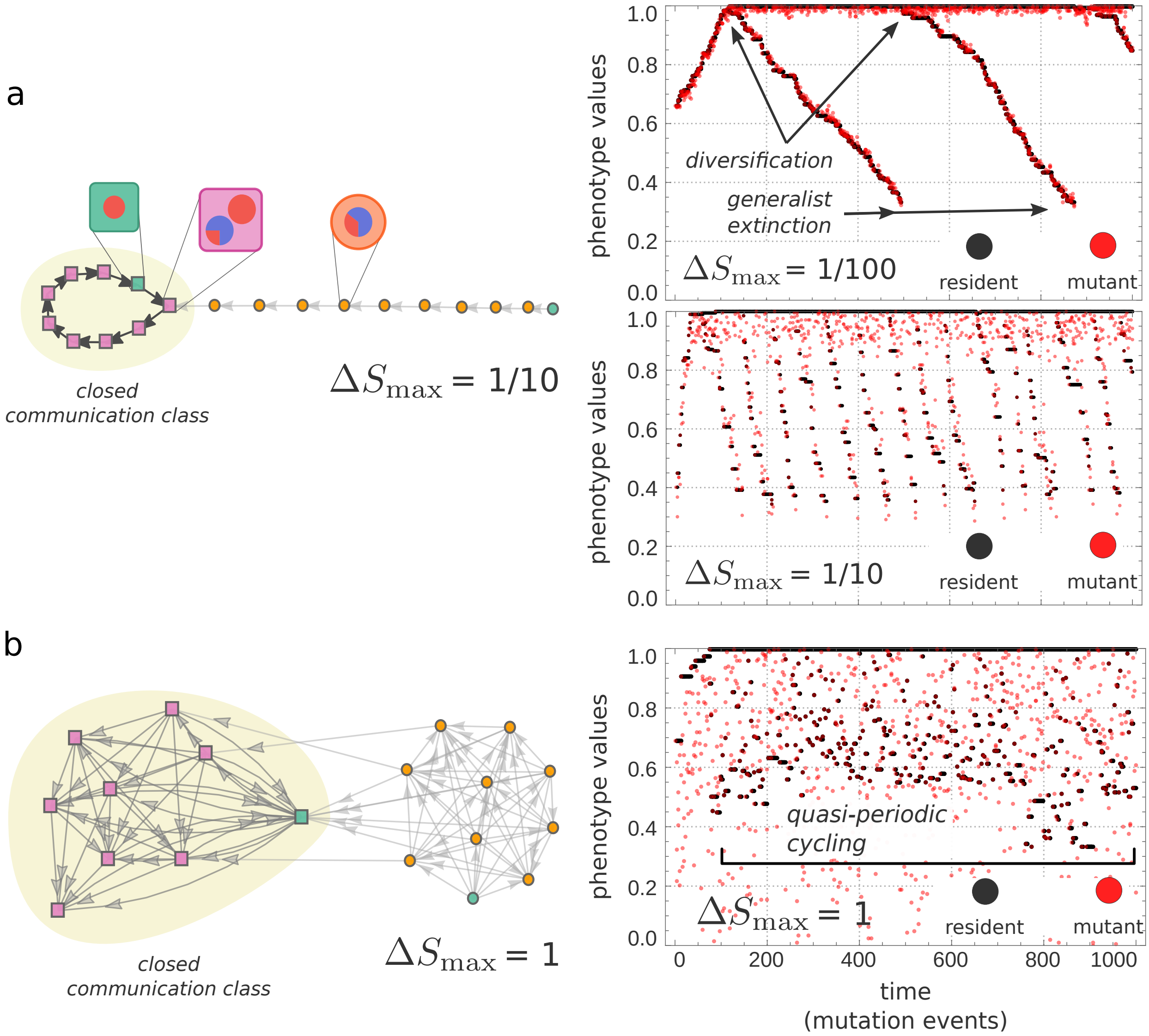

Supplementary Figure 3. **Comparing stochastic simulations in continuous phenotype space with simulations using a discrete phenotype space.** We illustrate agreement between the two simulation regimes for an example process that exhibits cycling and quasi-periodicity. (a) When the maximum mutation size is small ($\Delta S_{\max} < 1/10$), the process has a single periodic cycle of diversification and extinction. (b) For large mutations, the closed communication class in the Markov process is aperiodic.

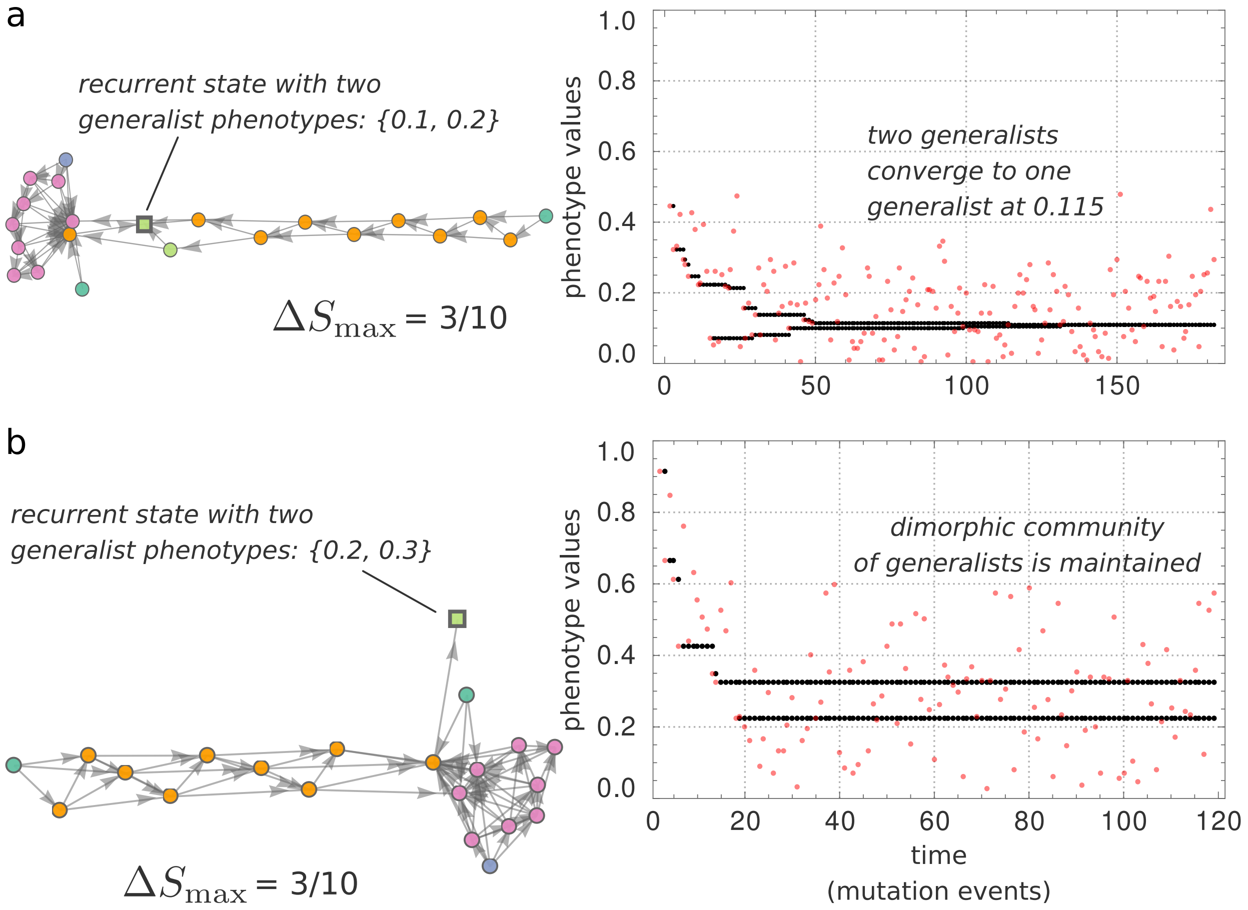

Supplementary Figure 4. **Stochastic simulations can cause dimorphic communities to converge to a monomorphic population.** Two co-existing populations can converge to a single monomorphic population if infinitesimally small mutations are permitted; discrete phenotype spaces, therefore, can in some cases allow dimorphic communities to be maintained. (a) In this process, the recurrent dimorphic community of generalists is not evolutionarily stable in the continuous case: the two phenotypes converge to a single generalist population. (b) In a different process, however, the recurrent dimorphic community of generalists is maintained in the continuous case.

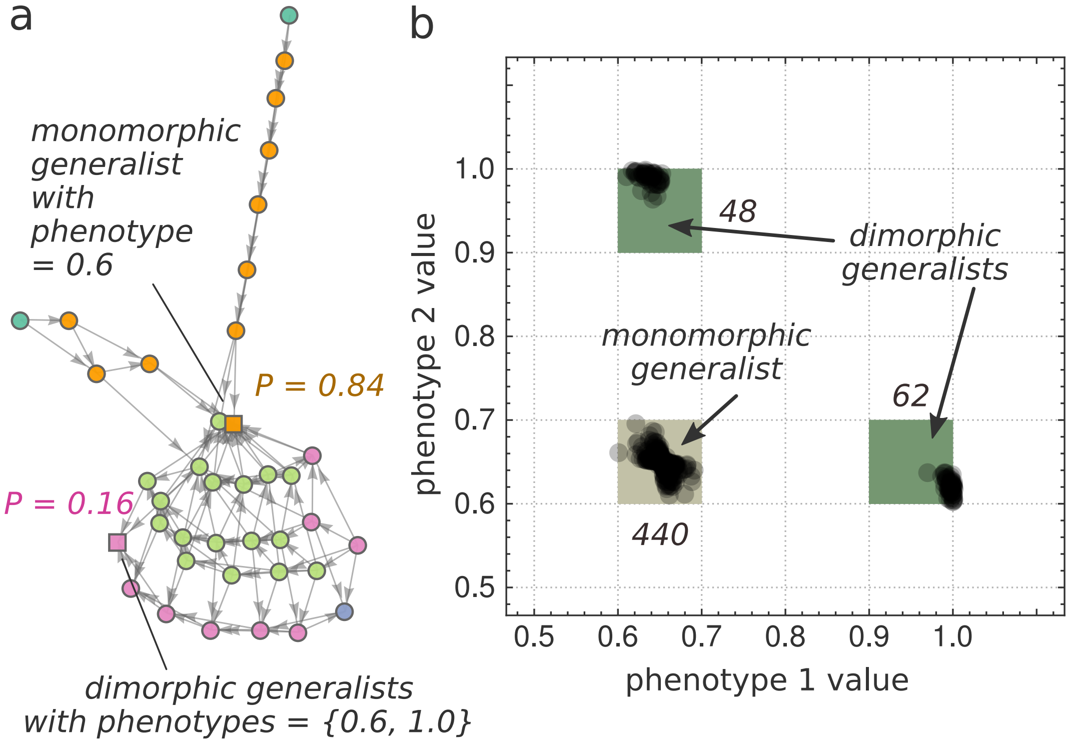

Supplementary Figure 5. **Stochastic simulations in continuous phenotype space for a multi-stationary process.** Stochastic simulations are in agreement with our usual deterministic simulations in a discrete phenotype space. (a) The network for the multi-stationary process with both a monomorphic and a dimorphic generalist as the recurrent states. The stationary probability for the monomorphic state is 0.84; the probability for the dimorphic state is 0.16. (b) Distribution of the end-points of 550 stochastic simulations in continuous phenotype space. Approximately 80% of mutational paths terminate around the monomorphic generalist state (phenotypes around $\{0.6, 0.6\}$); the remaining paths terminate at a hybrid state (phenotypes around $\{0.6, 1.0\}$ or $\{1.0,\ 0.6\}$).

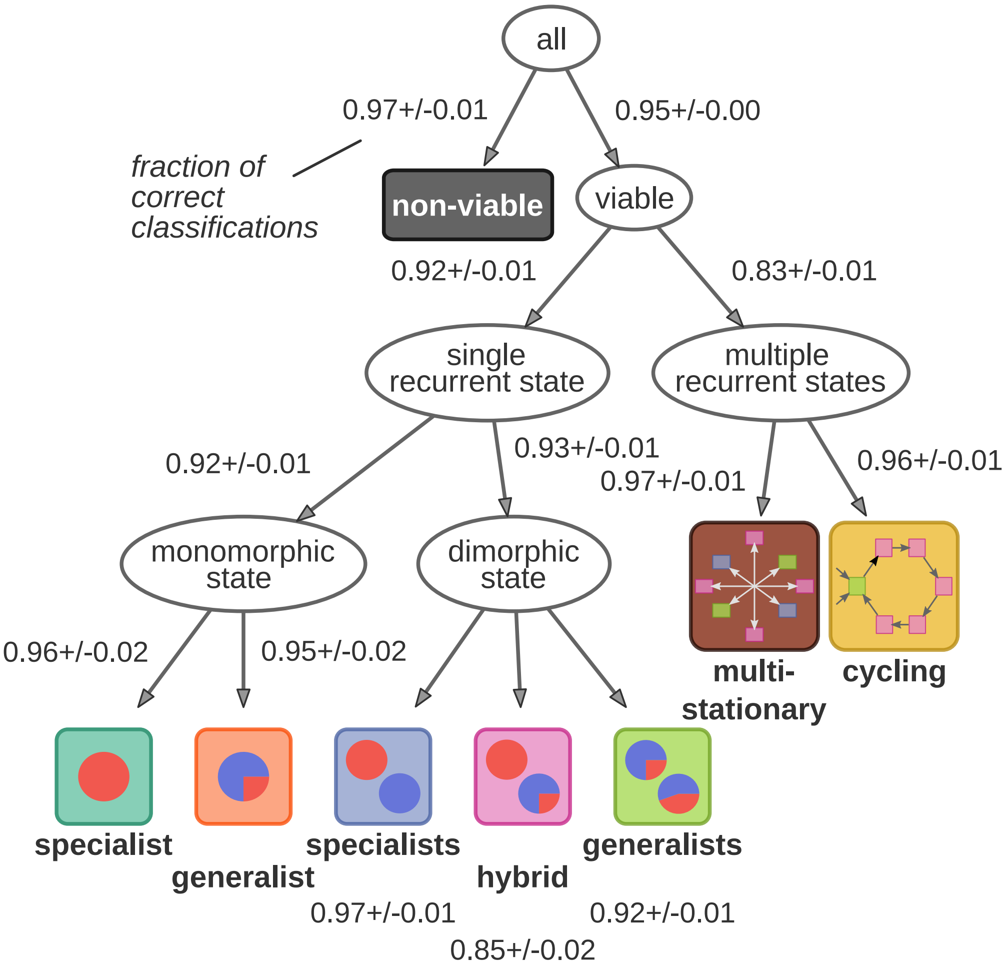

Supplementary Figure 6. A hierarchical classification model poorly predicts evolutionary outcomes from environmental parameters. The hierarchy of the classification model follows the hierarchical outcome scheme in Fig. 3a. Edges are labeled to indicate the node classifier recall (the fraction of relevant samples that were recovered) in a 10-fold cross-validation setting (reporting mean $\pm$ standard deviation). The hierarchical classifier's overall recall for each of the eight evolutionary outcomes is the product of the edges in the path from the root of the tree ('all' outcomes) to each of the eight leaves. For example, the recall for dimorphic generalists is $0.95 \times 0.92 \times 0.93 \times 0.92 = 0.75$. The average recall over the eight outcomes is 0.78, which suggests that predicting long-term outcomes from environmental conditions alone is likely to be difficult.

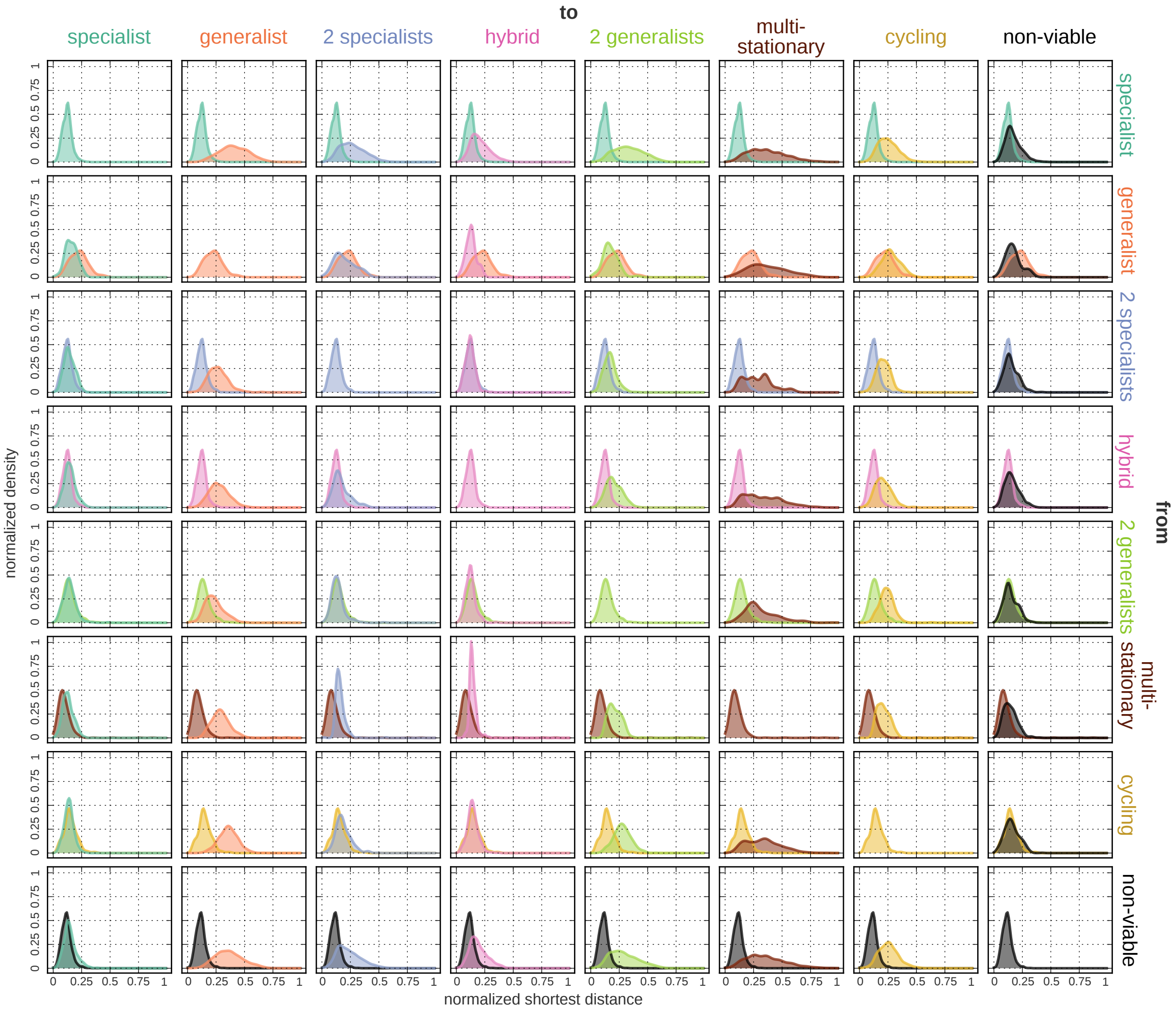

Supplementary Figure 7. **Shortest distance distributions between all pairs of evolutionary outcomes.** To construct the distributions, we determined for each point in a standardized parameter space the smallest Euclidean distance to another point of each of the eight evolutionary outcome classes (Methods). The i, j panel (counting from the top-left corner) shows the shortest distance distribution from the i'th outcome to the j'th outcome (and also, as a reference, to itself). The distributions are asymmetric, and the asymmetry arises because of differences between the shapes of the outcome clusters in parameter space (Fig. 4b).

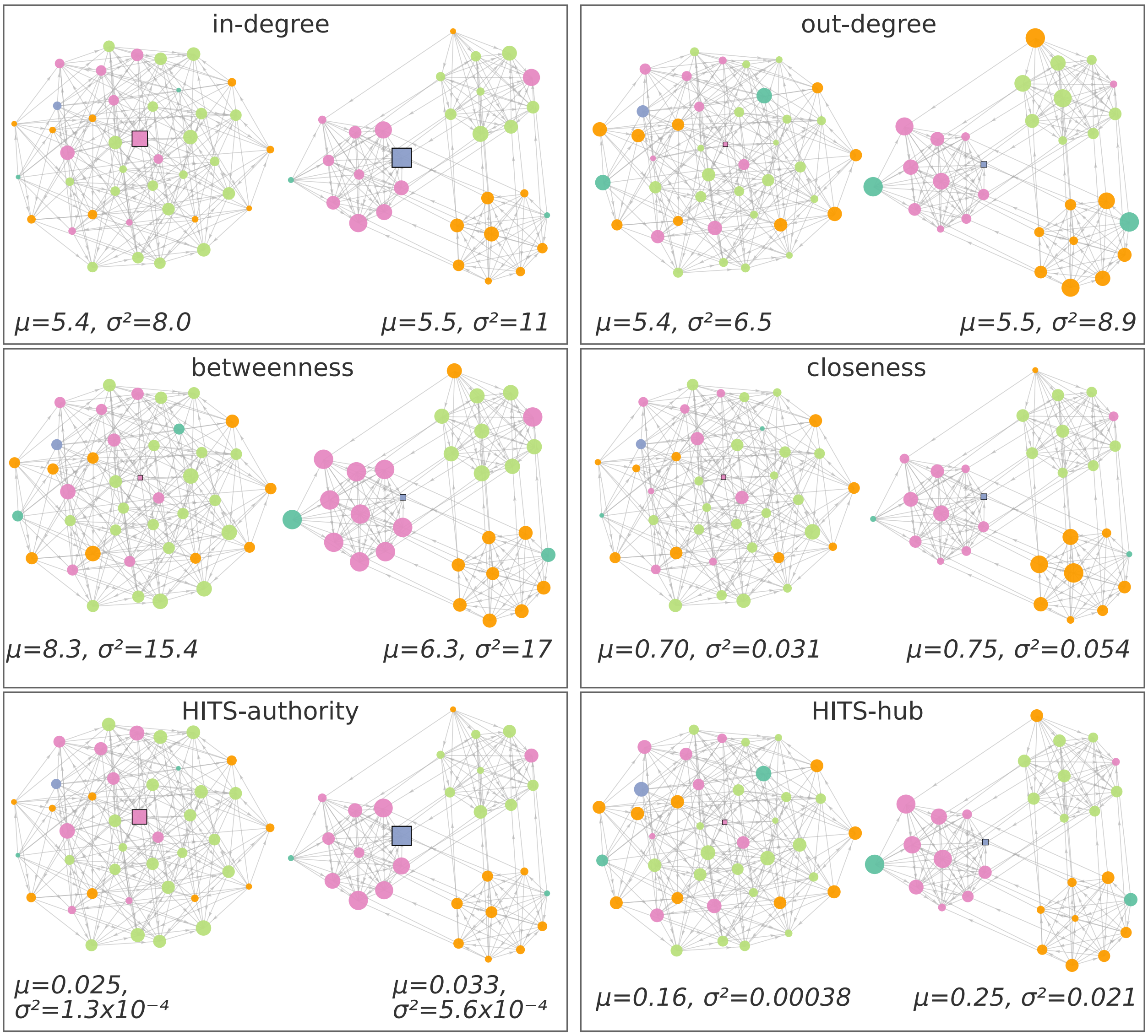

Supplementary Figure 8. **Illustration of the six network centrality measures**. To show how network vertices rank in terms of each centrality measure, we scaled the size of each vertex in proportion to its centrality value on a per-network basis. Brief descriptions of the centrality measures are given in Supplementary Table 1. The reported mean and variance, which we ultimately use to reduce the dimensionality of feature space for predictive models, is taken over the vertices in each network.

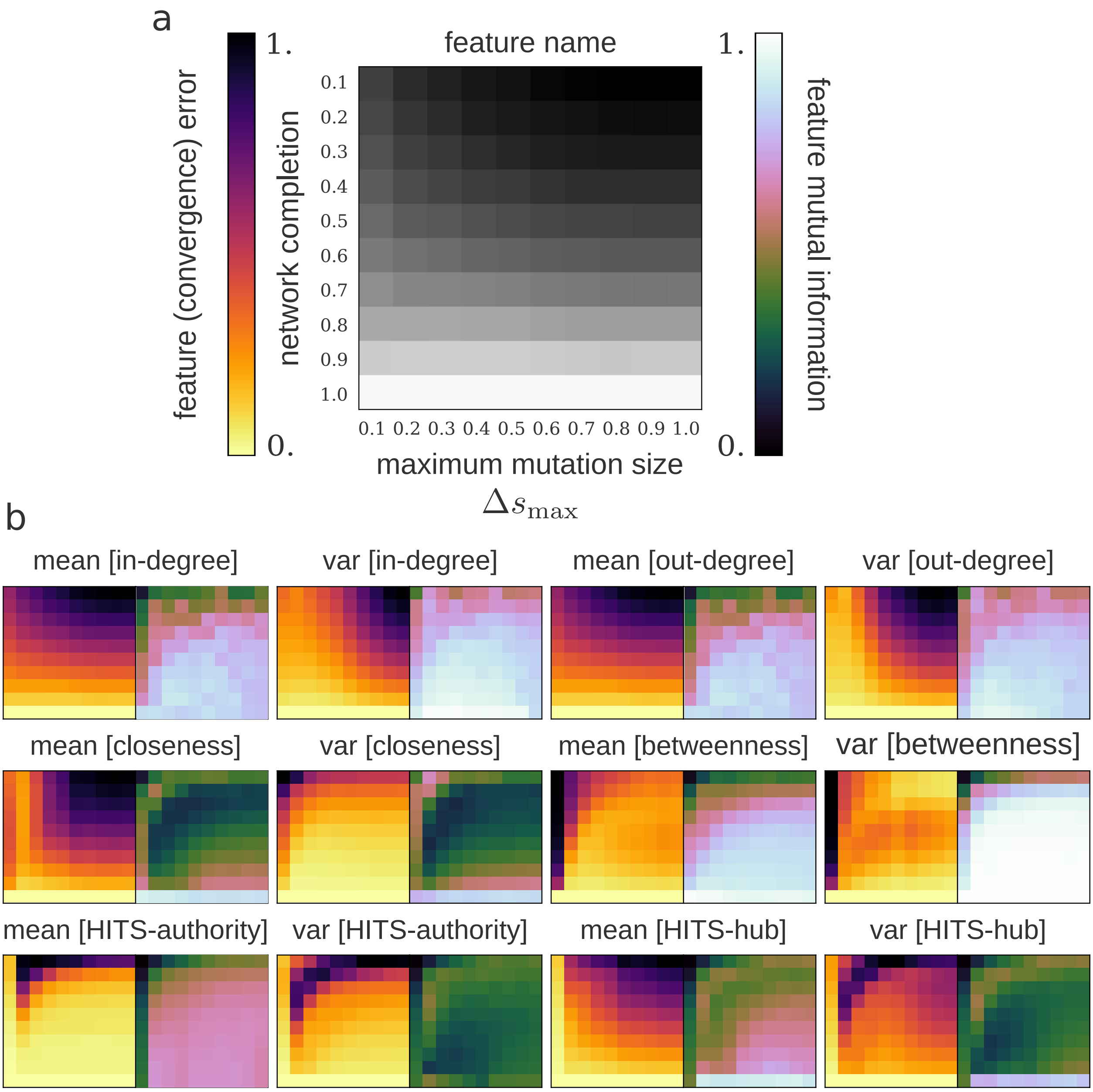

Supplementary Figure 9. Mutual information and (convergence) error for the mean and variance of six centrality measures. We show both mutual information and convergence error as a function of the fraction of network completion and the maximum mutation size (Fig. 6b). High mutual information implies that the centrality statistic can be used to make reliable predictions of the long-term evolutionary outcome because networks with different outcomes have distinct distributions of that centrality statistic. The convergence error is the difference between the statistic value of an incomplete network and its the statistic value for the same network's complete form (Methods). A low error implies that the incomplete network has centrality values similar to its complete network, which in turn implies that the incomplete network's topological properties have converged to those of its (ultimate) complete network. (a) Figure legend. Note that the network completion and maximum mutation size axes are transposed relative to Fig. 6. (b) The twelve centrality statistics characterized by both their mutual information with the evolutionary outcome and their convergence error.

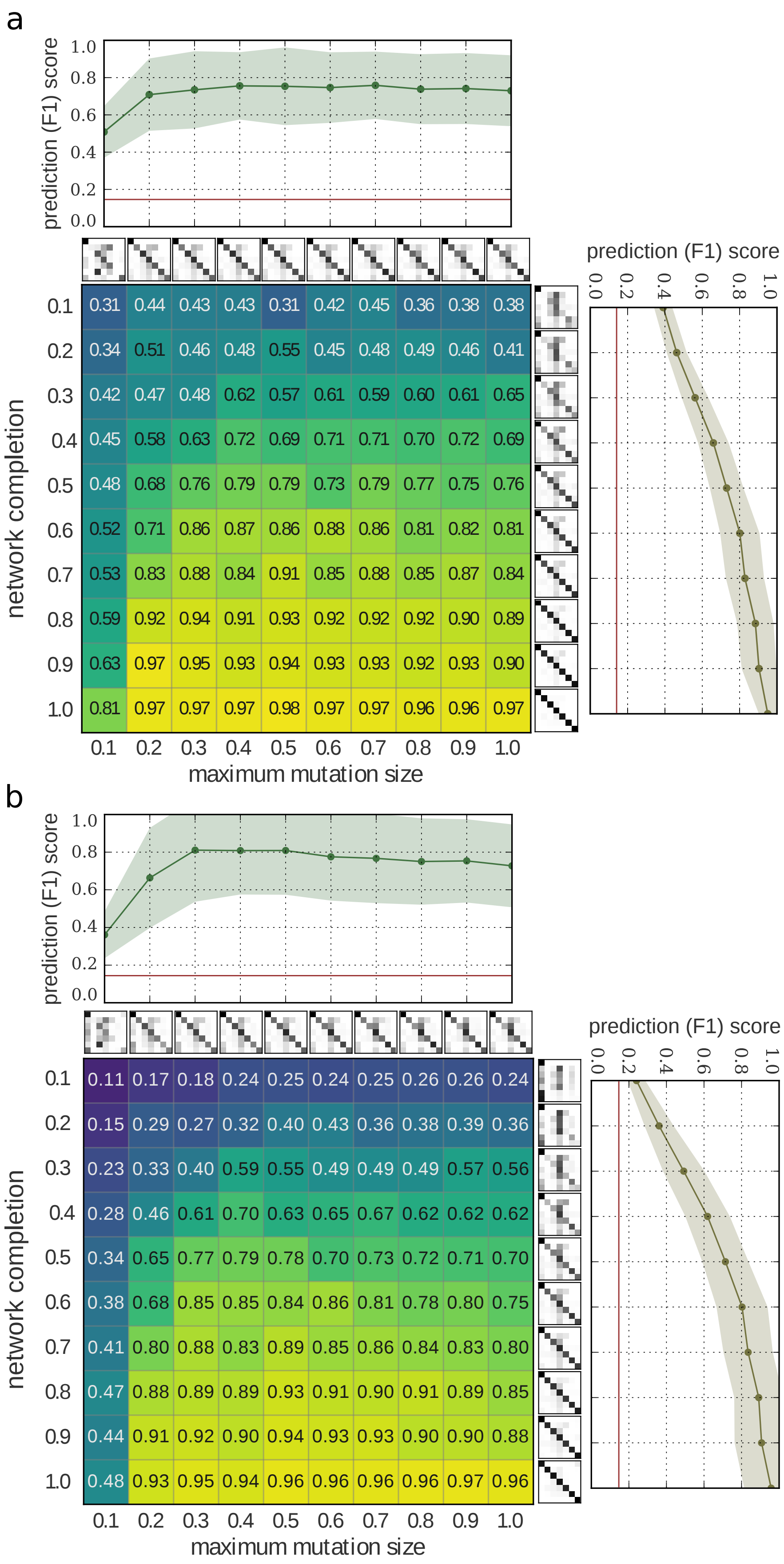

Supplementary Figure 10. Effect of marginalizing over the maximum mutation size and network completion during prediction. We assessed classifier performance via the mean unweighted $F1$ score taken over the seven evolutionary outcomes and report the mean test set performance in ten-fold cross-validation.

In both (a) and (b): *Top* and *right* line plots show average performance with the standard deviation in shaded regions taken over either the columns (network completion) or rows (maximum mutation size) of the central panel. The red line is the benchmark performance of a naive classifier that predicts following the empirical frequencies of the outcome classes. The 20 small insets show averaged confusion matrices for the seven evolutionary outcomes. (a) Network completion and maximum mutation size are known features during testing. (b) Network completion and maximum mutation size are 'missing' features during testing, and we marginalize over both to obtain prediction probabilities. This figure is a reproduction of Fig. 6c, with horizontal and vertical axes transposed, to allow easier comparison with Supplementary Fig. 10a.

Supplementary Table 1. Descriptions of the six centrality measures calculated for complete and incomplete networks.

**In-degree**	The number of edges arriving at a vertex. This number generally increases with increasing maximum mutation size so the variance between the vertices' in-degree is usually a better measure for comparing networks. From a biological point of view, a vertex's in-degree is the number of community states that can be transformed to the target state through a single mutation and invasion event.
**Out-degree**	The number of edges leaving a vertex. As with in-degree, the variance is a better measure for comparison. Biologically, it is the number of mutant phenotypes that can successfully invade the microbial community.
**Betweenness**	A measure of the number of shortest paths that pass through a vertex. That is, for a vertex v, its betweenness is $\sum_{s \neq v \neq t} \sigma_{st}(v)/\sigma_{st}$, where σ_{st} is the number of all shortest paths between vertices t and s, and $\sigma_{st}(v)$ is the number of those shortest paths that pass through v. For example, in the Supplementary Fig. 11 ('betweenness' panel, right-hand network) the cluster of pink (dimorphic hybrid) vertices have high betweenness because all paths, including shortest paths, must pass through these vertices to reach the recurrent state. A high betweenness indicates potential bottleneck points in the adaptation process, particularly when mutations can only be small, because many mutational paths must pass through the vertex with high betweenness.
**Closeness**	The reciprocal of the average geodesic (shortest) distance from a vertex to all other vertices. Vertices that have a large closeness can reach other vertices in fewer steps compared to vertices with low closeness. Potentially, vertices with high closeness indicate points in the adaptation process with high potential for diversification because they can transition to many other community states after a few mutation events.
**HITS-authority & HITS-hub**	The HITS algorithm assigns a high hub value for vertices that connect to other vertices with high authority value, and a high authority value to vertices that are connected to by vertices with a high hub value. Because of the cyclical nature of these definitions, the algorithm iteratively updates hub and authority values to determine each vertex's final values. Extending the definition to adaptation process, vertices with high authority values represent microbial communities that can be jointly reached by many other communities (the hubs) through mutation. The hub communities themselves can be invaded by many mutant populations to establish those communities (the authorities) that act as states commonly observed in parallel evolutionary experiments.
